# The Performance of *In Silico* Prediction Tools for Variant Curation in a Panel of Cancer Genes

**DOI:** 10.1155/humu/8817550

**Published:** 2026-06-19

**Authors:** Niles Nelson, Aram Niaz, Kirsten Fairfax, Tracy M. Bryan, Sionne Lucas, Joanne Dickinson

**Affiliations:** ^1^ The Menzies Institute for Medical Research, College of Health and Medicine, The University of Tasmania, Hobart, Tasmania, Australia; ^2^ Department of Molecular Haematology, The Royal Hobart Hospital, Hobart, Tasmania, Australia; ^3^ Children′s Medical Research Institute, Faculty of Medicine and Health, University of Sydney, Sydney, New South Wales, Australia, sydney.edu.au

## Abstract

Rare single base pair changes in genes are an important cause of disease, as they can reside in key regions of the gene influencing biological function by impacting the protein conformation and protein interactions. Generation of the necessary experimental evidence to define the outcome of the presence of these gene variants is time‐consuming and costly. These challenges have led to the development of a plethora of *in silico* prediction tools. These tools frequently use similar sources of information and are trained on overlapping multigene ‘truth’ datasets. However, frequently there has been no quantitative validation of the performance of these *in silico* tools for individual genes. Here, we have applied the ClinGen Sequence Variant Interpretation Working Group′s recommended *in silico* score thresholds and AlphaMissense predictions to a set of predisposition gene variants with established pathogenicity/benignity. Of the genes assessed (*BRCA1*, *BRCA2*, *TP53*, *TERT* and *ATM*), when recommended thresholds were used, *in silico* tool predictions showed inferior sensitivity (< 65%) for pathogenic *TERT* variants and inferior sensitivity (≤ 81%) for benign *TP53* variants. AlphaMissense outperformed the other tools for *TP53* but did not improve predictive accuracy for *TERT* variants. This validation study highlights that *in silico* tool performance can be gene‐specific and is dependent on the ‘training set’ on which the algorithm is built. Where there are sufficient numbers of established benign and pathogenic missense variants based on clinical and functional evidence, the use of *in silico* tool scores should be validated for individual genes. For genes where this is not possible and gene‐agnostic *in silico* score cut‐offs are used, consideration of missense variant–protein structural impact relationships is suggested.

## 1. Introduction

The interpretation of missense variants detected in cancer predisposition genes remains a significant challenge both for those in the clinical and research settings. Pathogenic variants can be ultrarare or even private to individual families, making enrichment in cases difficult to establish [1]. Phenotypes are often late‐onset, incompletely penetrant and nonpathognomonic, resulting in limited utility of segregation evaluation [1, 2]. Where affected family members are identified, it is also likely to be posthumously, therefore hampering segregation testing. Although functional genomic assays are available to permit assessment of pathogenicity/benignity for some genes (*TP53* [3, 4], *BRCA1* [5, 6], *BRCA2* [7] and *TERT* [8]), for many genes there remains a dearth of assays addressing function. The significant cost in terms of assay development, the labour intensiveness and the low throughput associated with functional genomic studies also impact the practical application for variant curation. Use of the American College of Medical Genetics and Association for Molecular Pathology (ACMG/AMP) criteria to permit formal curation of the variants to inform clinical decisions is therefore problematic, as for the vast majority of rare missense variants identified, the table of criteria cannot be applied [2, 9].

*in silico* tools provide an opportunity to assist with the interpretation of the likely pathogenicity of rare variants both in research and clinical settings. The ACMG/AMP criteria PP3 and BP4 were established to assist with the use of *in silico* tools for diagnostic curation [9]. The PP3 criterion is applied when *in silico* data predict that a variant is pathogenic, whereas the BP4 criterion is applied when *in silico* data predict that a variant is benign. As gene‐specific curation guidelines have developed, variant curation working groups have provided gene‐specific recommendations regarding the use of *in silico* prediction tools [1, 10]. Although ideally, *in silico* tools should be validated for individual genes, in reality, this is challenging. For many genes, there are insufficient established pathogenic and benign missense variants for validation of these tools [11]. This has resulted in some gene‐specific curation guidelines (*APC*, *ATM* and *CDH1*) relying on score thresholds determined by algorithm training on aggregated data from multigene ‘truth sets’ and some (e.g., *PALB2*) omitting the use of *in silico* tool scores altogether [12–15].

Evaluation of the performance of *in silico* tools has shown the potential to improve the curation of missense variants by adjusting the score thresholds to allow higher confidence in pathogenic or benign predictions [16, 17]. This study aims to assess the performance of these gene‐agnostic recommendations when applied to individual genes harbouring rare variants contributing to solid organ and/or blood cancer/inherited bone marrow failure risk. In addition to evaluating predictive performance of the *in silico* tools, REVEL [18], MutPred2 [19], Bayesdel (no AF) [20], VEST4 [21] and CADD [22, 23], we examined the impact of the recommended thresholds on predictive performance and whether consideration of protein structure (MISCAST [24] and AlphaMissense [25]) can improve prediction accuracy.

## 2. Material and Methods

To examine the performance of *in silico* tools in predicting pathogenicity, we examined the thresholds imposed to interpret the prediction scores generated by these tools (Table 1) [16]. Bayesian statistical analyses have been used to determine associated target positive likelihood ratios (PLRs) [26, 27]. It is estimated that in the clinical setting, diagnostic genomic testing will reveal a likely pathogenic variant in approximately 10% of the variants identified. This underlying estimate of prior probability (0.1) has been used to inform application of the ACMG/AMP criteria to deliver curated scores for pathogenicity. This has equated to PLRs (defined as an OddsPaths) of greater than 2.1, 4.3, 18.7 and 350 to achieve the thresholds of ‘supporting’, ‘moderate’, ‘strong’ and ‘very strong’ strength levels, respectively [27]. The final posterior probability associated with this approach to curation is estimated to be > 0.9 for a likely pathogenic curation and > 0.99 for a pathogenic curation. The inverse of this applies to benign and likely benign curations.

**Table 1:** *in silico* tool score cut‐offs used in this study (derived from Pejaver et al., 2022).

*in silico* tool	Pathogenic (PP3)			Benign (BP4)		
	Strong	Moderate	Supporting	Supporting	Moderate	Strong
REVEL	≥ 0.932	[0.773; 0.932)	[0.644; 0.773)	(0.183; 0.290]	(0.016; 0.183]	(0.003; 0.016]
BayesDel	≥ 0.50	[0.270; 0.500)	[0.130; 0.270)	(−0.360, −0.180]	≤ −0.360	—
VEST4	≥ 0.965	[0.861; 0.965)	[0.764; 0.861)	(0.302; 0.449]	≤ 0.302	—
MutPred2	≥ 0.932	[0.829; 0.932)	[0.737; 0.829)	(0.197; 0.391]	(0.010, 0.197]	≤ 0.010
CADD	—	≥ 28.100	[25.300; 28.100)	(17.300; 22.700]	(0.150; 17.300]	≤ 0.150

### 2.1. Selection of *In Silico* Tools

The following tools were selected for analysis in this study: REVEL [18], MutPred2 [19], Bayesdel (no AF) [20], VEST4 [21] and CADD [22, 23]. *In silico* tool selection was based on the breadth of evidence strength determined by Pejaver et al. 2022. Tools were prioritised that potentially provided up to a strong level of evidence to meet the ACMG criteria for PP3 or BP4.

REVEL, a random forest classifier, integrates functional impact prediction tool results (MutationAssessor [28], MutationTaster [29], PolyPhen‐2 [30], LRT [31] and SIFT [32]), conservation score tool results (GERP++ [33], PhyloP [34] and SiPhy [35]), predictions from other meta‐predictors (FATHMMv2.3 [36], PROVEAN [37] and VEST3 [38]) as well as protein domain and population allele frequency data for a given missense variant to predict pathogenicity [18]. MutPred2 utilises a deep neural network trained on curation data (ClinVar [39] and HGMD [40]), interspecies pairwise alignments, population allele frequency data (ExAC [41]) and protein structural and functional data (UniProt [42] and the Protein Data Bank [43]). BayesDel (no AF) is also an ensemble tool based on a native Bayes classifier trained on curation data (ClinVar and HGMD), the same functional impact and conservation tools utilised for REVEL. Variants were also annotated with FATHMM predictions. Population allele frequency data were intentionally not included to assist with rare variant interpretation. VEST4, also a random forest classifier, utilises disease (HGMD) and population (ESP6500 [44]) data and SNVBox [45] annotations to generate predictions. Although similar conservation and functional data usage is similar (Grantham, Polyphen, SIFT and others), CADD is unique amongst these tools, in that the underlying algorithm was not trained on training sets of known disease‐causing gene variants with established non–variant of uncertain significance (VUS) classifications. CADD also integrates some splice prediction into the scoring algorithm [20, 22]. AlphaMissense is an additional *in silico* tool that has been developed subsequent to the above‐described analysis; however, the initial validation analysis is suggestive that it may outperform many of these other established tools [25]. The variants in the included datasets were annotated with AlphaMissense scores (Tables S1–S7) as a comparison to the tools reviewed by Pejaver et al. (2022). The recommended cut‐offs of ≥ 0.564 for PP3 and ≤ 0.34 for BP4 were used for all genes with the additional assessment of ≥ 0.75 for PP3 and ≤ 0.65 for BP4 for *BRCA1* based on a gene‐specific validation [46]. In this study, application of AlphaMissense data for PP3/BP4 was assessed at a supporting level only.

All missense variants classified as benign or likely benign included in the analysis were screened using a SpliceAI [47] threshold of ≥ 0.2 to assess the risk of creation of a cryptic donor or acceptor site (Tables S16 and S17). This cut‐off threshold was determined based on current usage in guidelines [2, 48–50].

### 2.2. MISCAST to Predict Pathogenicity Using Protein Structural Impact Prediction

*In silico* tool algorithms often incorporate predictions regarding the structural impact of missense variants on the translated protein. As it is not possible to determine how these predictions are incorporated into *in silico* algorithms, the influence of these predictions on an *in silico* score for a given missense variant is difficult to determine. MISCAST [24] is an *in silico* tool that incorporates only structural impact predictions for missense variants, which can give insight into the impact of these predictions on the overall *in silico* score for a given missense variant. The algorithm used to generate MISCAST predictions (P3DFi scores) was trained using similar gene variant reference sets as many of the commonly used *in silico* tools [18–20]. To develop the MISCAST algorithm, variants from 1330 disease‐causing genes were extracted from the ClinVar and the HGMD databases and compared to gene variants detected in healthy population controls (data extracted from gnomAD) to identify protein structural impact features that are enriched in disease‐causing variants [24]. To identify if MISCAST predictions could predict pathogenicity for the gene variants included in this present study, variant summary reports were downloaded from MISCAST [24] for *TERT*, *TP53*, *BRCA1*, *BRCA2* and *ATM* and annotated with the benign and pathogenic classifications from the truth sets discussed below. The proportion of pathogenic and benign variants was determined for each ‘all protein classes’ P3DFi score as described previously [24]. The benign variant proportion was subtracted from the pathogenic variant proportion for the P3DFi scores < −2, −2, −1, 0, 1, 2 and > 2, respectively. Result visualisation was generated using ggplot2 (https://ggplot2.tidyverse.org). Code is available on request.

### 2.3. Generation of ‘Truth Sets’ of Missense Variants for Rule Evaluation

In order to evaluate the positive predictive value and negative predictive value of an *in silico* score cut‐off, it must be tested on a set of known pathogenic and known benign variants: a truth set. Genes were considered where gene‐disease validity (GenCC) is established for solid tumour predisposition (*BRCA1*), haematological malignancy/bone marrow failure (*TERT*) predisposition or both [51]. For many cancer predisposition genes, there are insufficient numbers of variants with definitive pathogenic or benign classifications to create a truth set large enough for statistical analysis. Using the bootLR R package [52] (see below) for an *in silico* tool that was 97% sensitive and 97% specific, a truth set would need at least 30 pathogenic and 31 benign variants to be sufficiently powered to assess a moderate strength level target (PLR > 4.3). Therefore, the genes *TP53*, *BRCA1*, *BRCA2*, *ATM* and *TERT* were selected as having more than 61 missense variants with benign or pathogenic classifications available for each gene on public variant curation databases. The ClinGen Evidence Repository of variants curated by variant curation expert panels was also used as a source of established benign and pathogenic variants [53, 54]. For the purposes of this study, pathogenic and likely pathogenic variants were grouped together and considered as pathogenic and similarly, benign and likely benign variants were both considered benign. Only germline variants associated with germline cancer predisposition were included in the analysis. A truth set was defined as a set of variants that have not been classified as a VUS as determined by the curation criteria applied in the relevant source database.

#### 2.3.1. *TP53*

Two *TP53* truth sets were used in this analysis. The first *TP53* missense variant truth set was obtained from Fortuno et al., 2018 [55]. Pathogenicity and benignity were determined based on variant allele frequency in gnomAD, dominant negative effect and results from transactivation assays. Of the 268 variants that were available for analysis, 247 were pathogenic, and 21 were benign. REVEL scores and BayesDel (no allele frequency) scores were obtained from the publication [55]. VEST4, CADD and MutPred2 scores were obtained from the dbNSFP (Version 4.2) database of *in silico* tools on 11 August 2022 (Table S1) [56]. Following variant data upload, output of *in silico* results for 269 variants (248 pathogenic and 21 benign) was available for further analysis. The second truth set (TP53_2) used was derived from the *TP53* variants included in the ClinGen Evidence Repository truth set discussed below (Table S2) [53, 54]. Pathogenic variants were included if ACMG/AMP criteria for likely pathogenic or pathogenic were met without the use of PP3. Benign variants were included if at least one benign strong level criterion was met and no conflicting pathogenic moderate, strong or very strong criterion was met.

#### 2.3.2. *BRCA1* and *BRCA2*

*BRCA1* and *BRCA2* missense variants were obtained on 16 July 2022 from the *BRCA1* and *BRCA2* Mutation Databases (University of Utah). Variant classification methods are described in the associated publication [57]. Briefly, gene‐specific PLRs were estimated for co‐occurrence with known deleterious variants, phenotypic and segregation data. Variants with combined odds of 1:100 and 1:1000 were considered likely pathogenic and pathogenic, respectively [58]. For the *BRCA1* gene, 44 pathogenic and 267 benign missense variants were available for assessment. Following upload to the dbNSFP (Version 4.2) database on 11 August 2022, *in silico* output (REVEL, VEST4, MutPred2, BayesDel [no allele frequency] and CADD) results for 43 pathogenic and 256 benign variants were obtained. *in silico* results were available for all 20 pathogenic and 286 benign *BRCA2* missense variants that were extracted (Tables S3 and S4).

#### 2.3.3. *ATM*

*ATM* missense variants were obtained from the Leiden Open Variation Database (LOVD3) on 16 July 2022 [59]. Variants, as well as associated clinical and published data, are submitted to LOVD3 from users at institutions with local LOVD3 instances. Variant curations (clinical classifications) are determined by the assigned LOVD3 curator. Forty pathogenic and 35 benign variants were extracted. *in silico* results were obtained for 36 and 33 variants, respectively (Table S5).

#### 2.3.4. *TERT*

The TERT_1 dataset comprises missense variants meeting ACMG/AMP criteria for pathogenicity or benignity without the use of the PP3/BP4 criterion. This dataset was derived from a comprehensive review and curation of all known *TERT* variants reported in the literature as well as the ClinVar and gnomAD databases. Details regarding the application of ACMG/AMP guidelines are described elsewhere [8]. Thirty‐two variants with a pathogenic classification and only six variants with a benign classification were identified (Table S6). As reported previously, only a small minority of missense variants reported for *TERT* can be established as pathogenic or benign using current ACMG/AMP guidelines [8]; thus, this dataset was underpowered to assess PLRs for PP3/BP4 at moderate strength levels. Therefore, sensitivity analysis was also undertaken using two related truth sets: TERT_2 (Table S6) and TERT_3 (Table S7). Details regarding these truth sets are included in the Supporting Information tables.

#### 2.3.5. ClinGen Data

Missense variants were also extracted for each of the genes from the ClinGen Evidence Repository on 16 July 2022 [54]. The ClinGen Evidence Repository provides detailed and standardised variant curation information for all included variants. Seven hundred and thirty‐eight pathogenic and 361 benign missense variants from 50 genes were available for extraction (Table S8). *In silico* results were obtained from 721 and 352 variants, respectively. A second truth set of ClinGen variants was also assessed to attempt to control for variant classifications dependent on use of *in silico* tool results. In this truth set, pathogenic variants were considered as variants that met ACMG/AMP guidelines for likely pathogenic or pathogenic without the use of PP3. Benign variants were considered as variants where a benign code could be applied at a strength level of strong, very strong or stand‐alone. This truth set resulted in 629 pathogenic and 384 benign variants with *in silico* tool scores available for evaluation.

All variants extracted from the curation databases described above were converted to hg19 genomic coordinates using Mutalyzer (Version 3) [60]. Data were then uploaded to the dpNSFP (Version 4.2) [56] database in a bed file format.

### 2.4. Receiver Operating Characteristic (ROC) Curve

The receiver operating characteristic (ROC) curve were generated using the plotROC R package [61]. The melt_roc function was used to combine results from each *in silico* tool into single graphs.

### 2.5. Sensitivity, Specificity and Positive Predictive Value and Negative Predictive Value Analyses

For each tool, true positive, true negative, false positive and false negative results were determined for each cut‐off (Table 1). For the assessment of PP3, variants classified as pathogenic were considered as true positives and false negatives if the associated *in silico* tool scores fell above or below the defined cut‐off, respectively. False positives and true negatives were defined as variants with a benign classification and an *in silico* tool score that fell above or below the defined cut‐off, respectively. This assessment was performed at all strength levels provided for each *in silico* tool assessed. For the assessment of BP4, variants classified as benign were considered as true positives and false negatives if the associated *in silico* tool scores fell below or above the defined cut‐off, respectively. False positives and true negatives were defined as variants with a pathogenic classification and an *in silico* tool score that fell below or above the defined cut‐off, respectively.

Sensitivity = true positives/(true positives + false negatives)

Specificity = true negatives/(true negatives + false positives)

PPV = true positives/(true positives + false positives)

NPV = true negatives/(true negatives + false negatives)

Table 1 indicates estimated threshold ranges based on those established by Pejaver et al. for all tools assessed in this study: ‘[’ indicates that the range includes the end value; ‘(’ indicates that the range excludes the end value. An ‘—’ indicates that the tool was unable to meet the posterior probability threshold. Analyses of the tools BayesDel and VEST4 in the Pajaver et al. (2022) study did not show cut‐off scores that reached a posterior probability sufficient for the application of BP4 at a strong level. Given this, these tools were not assessed for BP4 strong‐level evidence in this study. This was also the case for CADD and PP3 strong‐level evidence.

### 2.6. Generation of PLRs

Positive likelihood ratio = Sensitivity/(1 − Specificity).

Approximately 1/3 of tests (37%) were associated with 100% specificity, impacting the ability to calculate meaningful confidence intervals. To address this, confidence intervals were calculated using a bootstrapping method described elsewhere [52]. Briefly, the lowest population specificity that could result in a sample size specificity of 100% is calculated using the formula *p* = *e* ^1/*n*^(log 0.5). The average result from 50 tests of 10,000 bootstrap samples drawn from a parametric binomial distribution is then used to estimate the confidence interval. The R package bootLR with the following arguments was used to perform these calculations.

BayesianLR.test(TPo, TPa, TN and TB)

TPo = true positives

TN = true negatives

TPa = total pathogenic

TB = total benign

## 3. Results

**Figure 1:**
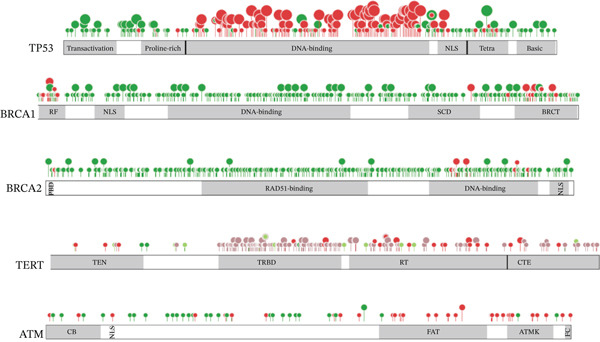
Location of pathogenic and benign variants assessed for *TP53*, *BRCA1*, *BRCA2*, *TERT* and *ATM*. Pathogenic and likely pathogenic variants are represented in red, and likely benign and benign variants are represented in green (TERT_1). Variants suspicious for pathogenicity and benignity (TERT_2) are coloured pink and pale green, respectively. The number of unique variants reported at a single codon is represented by the size and height of the lollipop symbol (1, 2, 3, 4 or 5 variants). Domain labels are as follows; NLS (nuclear localisation signal), Tetra (tetramerization), RF (ring finger), SCD (serine cluster domain), BRCT (*BRCA1* C‐terminal), PBD (*PALB2* binding domain, TEN (telomerase essential N‐terminal domain), TRBD (telomerase RNA binding domain), RT (reverse transcriptase domain), CTE (C‐terminal extension), CB (chromatin binding and substrate interaction), FAT (focal adhesion targeting domain), ATMK (ATM kinase domain), FC (FATC domain). Reference transcripts used are NM_000546, NM_007294, NM_00059, NM_196253 and NM_000051 for *TP53*, *BRCA1*, *BRCA2*, *TERT* and *ATM* plots, respectively. Retrieved from ProteinPaint [62].

The variants included in the analyses presented are represented in Figure 1. This diagrammatic representation demonstrates that the majority of pathogenic variants for *TP53* cluster in the DNA‐binding domain, whereas this is not evident for *BRCA1*. Notably, for *TERT* pathogenic variants appear to be distributed throughout the gene. In total, 1228 variants (593 pathogenic and 635 benign) in cancer predisposition genes and 1105 variants (721 pathogenic and 384 benign) in a ClinGen curated data set were used as a truth set to assess commonly used *in silico* tools. Results were available for 299, 306, 344, 69, 210 and 1105 variants for *BRCA1*, *BRCA2*, *TP53*, *ATM*, *TERT* and the ClinGen groups (respectively), and Figure 1 depicts the location of each of these variants within these genes of interest.

### 3.1. Sensitivity and Specificity

**Figure 2:**
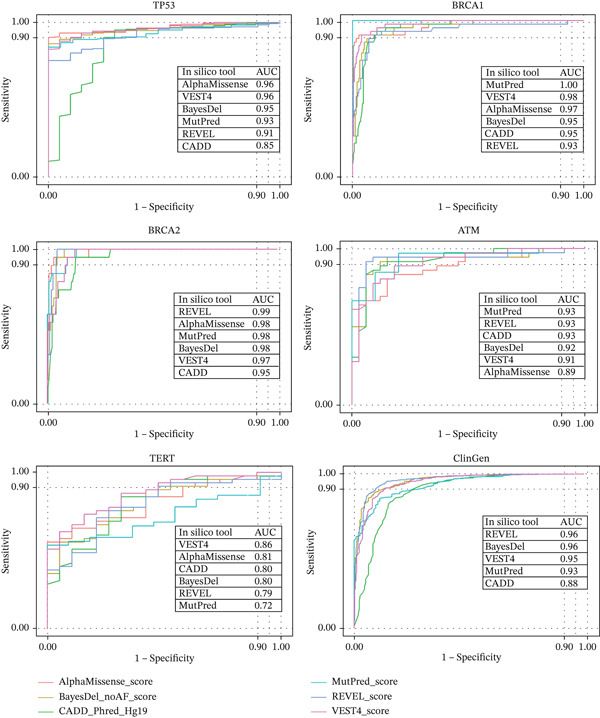
Receiver operating characteristic (ROC) curves showing comparative performance of *in silico* predictors for each gene included in this analysis as well as the combined dataset of gene variants curated by ClinGen VCEP committees. Area under the curve (AUC) measurements for each *in silico* tool are annotated for each combination ROC curve arranged in descending order. REVEL scores were associated with the greatest AUC for *BRCA2* and the ClinGen curated gene variants. AlphaMissense scores were associated with the highest AUC for *TP53* (TP53_1) variants. MutPred scores were associated with the highest AUC for *BRCA1* and *ATM*, and VEST4 was associated with the highest AUC for *TERT* (TERT_3) variants. ROC curve generated using the plotROC R package [61]. AUC measurements were calculated using the R package pROC [63].

**Figure 3:**
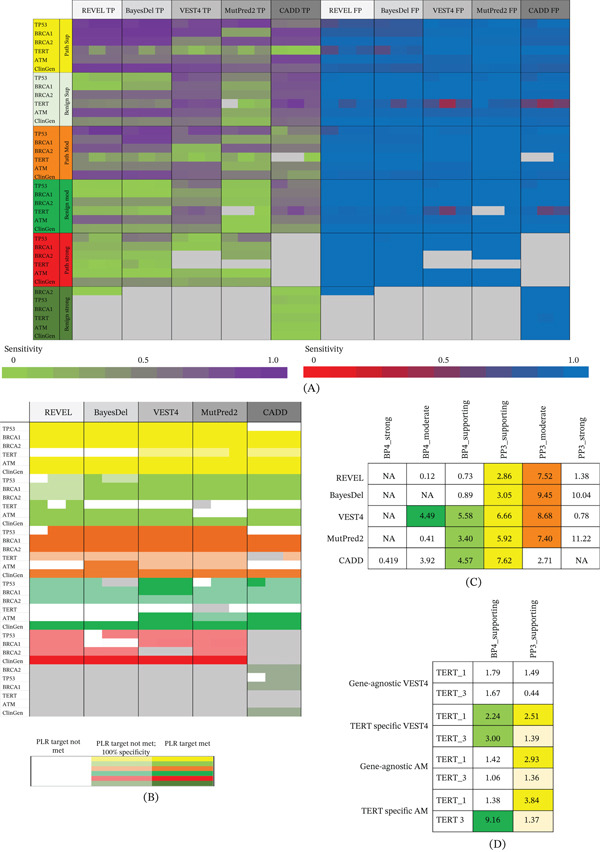
(A) Heatmap showing comparative true positive (TP) rate and false positive rate (FP) results for the included *in silico* tools at each evidence strength level cut‐off score. The rows reflect the truth set tested, and the columns reflect the *in silico* tool applied. Where there was more than one truth set used for a gene (such as for TERT), the row was further subdivided into each truth set in numerical order (1, 2 and 3) from left to right. Grey cells reflect *in silico* tool predictions that were associated with zero true positive results or *in silico* tool predictions that did not meet posterior probability targets [16] (see Section 2). (B) Heatmap showing comparative PLRs. The rows are the truth set tested, and the columns are the *in silico* tool. White represents *in silico* tool predictions that were associated with less than 100% specificity and a PLR that was below the target PLR for the given strength level. Where there was more than one truth set used for a gene (such as for TERT), the row was further subdivided into each truth set in numerical order from left to right. Three truth sets are included for *TERT*, and there are two truth sets for both *TP53* and the ClinGen curated variants. Where there was insufficient data to determine a PLR, this is represented in grey. (C) PLR 95% confidence interval lower limits for the *TP53* gene truth set TP53_2. Where PLR targets are met for PP3_supporting, PP3_moderate, PP3_strong, BP4_supporting, BP4_moderate and BP4_strong, this is represented by yellow, orange, red, light green, green and olive green, respectively. Where there was insufficient data to determine a PLR, this is represented with ‘NA’. Poor sensitivity of BP4 predictions for benign variants has impacted the PLR for REVEL and BayesDel for *TP53* variant predictions. (D) Comparison of PLRs generated for*TERT* from gene‐agnostic cut‐off scores for VEST4 and AlphaMissense versus cut‐off scores optimised for improved sensitivity. Bold yellow and green represent PLR 95% CI lower limits that were greater than the target of 2.1 for PP3 supporting and BP4 supporting, respectively. Pale yellow reflects a prediction associated with 100% specificity.

No single *in silico* tool was superior for all five genes reviewed in this analysis (Figure 2). Supporting‐, moderate‐ and strong‐level‐evidence cutoffs for each of the *in silico* tools are presented in Table 1. At the PP3 (multiple lines of computational evidence support a deleterious effect on the gene/gene variant) recommended supporting level cut‐offs, overall, excluding predictions for *TERT*, the tools were found to have a median sensitivity of 84%. Tool performance was poorest for *TERT* predictions, with a sensitivity of less than 65% for pathogenic *TERT* variants observed for all tools tested. In addition, MutPred predictions for *BRCA1* and ClinGen2, CADD predictions for *TP53* and ClinGen2, VEST4 predictions for *BRCA2* and REVEL, BayesDel and MutPred2 predictions for *ATM* showed a sensitivity of < 70%. For the PP3 moderate strength cut‐offs, only the REVEL predictions for *TP53* and *BRCA2*, the BayesDel predictions for *TP53* and *BRCA1* and the MutPred2 predictions for *TP53* delivered a sensitivity of greater than 70%. At the level of strong PP3 level of evidence, although predictive scores delivered high specificity, they were associated with very poor sensitivity at less than 40% with the exception of the BayesDel prediction for *TP53* and the ClinGen truth set (which had sensitivities of 57% and 42%, respectively; Figure 3A).

Tool specificity at the PP3 supporting level cut‐off was superior to the sensitivity (median, 91%). REVEL and BayesDel predictions for *TP53* and *TERT* were the most inferior (range, 67%–89%). Specificity was greater than 90% for the majority of predictions at the moderate strength cut‐offs, the REVEL prediction for *TP53* and the CADD prediction for *BRCA2* being the exceptions. Specificity at the recommended cut‐offs for PP3 strong‐level evidence was very high. Out of the 2113 total variants assessed, false positive calls at the strong‐level‐evidence cut‐offs were 7 (0.33%), 2 (0.09%), 2 (0.09%) and 1 (0.05%) for BayesDel, REVEL, MutPred2 and VEST4, respectively. BP4 results were further skewed towards high specificity (Figure 3A and Table S9). VEST4 and CADD were associated with the highest sensitivity for BP4 for the cut‐offs used.

### 3.2. PLRs for PP3

PLRs were determined for all *in silico* tools (Figure 3B). A prediction was considered to have met the target PLR if the lower limit of the 95% confidence interval met or exceeded the target suggested by the ACMG/AMP guidelines (2.1, 4.3 and 18.7 for supporting‐, moderate‐ and strong‐level evidence, respectively). At the cut‐off scores assessed (Table 1), all tools met or exceeded the target likelihood ratio of 2.1 for PP3 supporting‐level evidence with the CADD predictions for *TP53 (TP53_1)* and all predictions for *TERT* being the exceptions. No false positive calls were associated with the VEST4, MutPred2 and CADD supporting level cut‐offs for *TERT* but poor sensitivity (range, 4%–62%) resulted in 95% CI lower limits of less than 2.1. For all genes apart from *TERT* tested using the PP3 supporting level cut‐offs, MutPred2 predictions were associated with PLRs that met or exceeded the PP3_moderate target of 4.3.

*In silico* predictions less consistently met the PLR targets for PP3 moderate‐level evidence. The REVEL score predictions for *TP53* (TP53_1) failed to meet the PP3_moderate PLR target with a PLR of 3.7 (2.5; 6.19). The VEST4 and REVEL predictions for *ATM* were also slightly inferior to the target for PP3_moderate: 11 (3.658; inf) and 14.667 (3.875; inf), respectively. There were no false positive calls associated with the REVEL, BayesDel, MutPred2 and VEST4 predictions for *TERT(*TERT_1 and TERT_2); however, the sensitivity for pathogenic variants was poor; thus, the lower 95% CI limits were all less than 4.3. CADD performed the worst, with only the predictions for *BRCA1* and *BRCA2* exceeding the 4.3 target.

dictions less consistently met the PLR targets for PP3 moderate‐level evidence. The REVEL score predictions for *TP53* (TP53_1) failed to meet the PP3_moderate PLR target with a PLR of 3.7 (2.5; 6.19). The VEST4 and REVEL predictions for *ATM* were also slightly inferior to the target for PP3_moderate: 11 (3.658; inf) and 14.667 (3.875; inf), respectively. There were no false positive calls associated with the REVEL, BayesDel, MutPred2 and VEST4 predictions for *TERT(*TERT_1 and TERT_2); however, the sensitivity for pathogenic variants was poor; thus, the lower 95% CI limits were all less than 4.3. CADD performed the worst, with only the predictions for *BRCA1* and *BRCA2* exceeding the 4.3 target.

Although all PLRs exceeded the target for the cut‐offs recommended for PP3_strong, poor sensitivity resulted in the lower limit of the 95%CIs falling below the target of 18.1 for *TP53*, *BRCA1* and *BRCA2*. *TERT* and *ATM* had insufficient numbers of variants tested to assess a PLR target of 18.1. Use of CADD scores at a strong strength level has not been determined.

### 3.3. PLRs for BP4

To enable comparison, BP4 results have been determined using PLRs. Poor sensitivity resulted in *in silico* tools inconsistently meeting target PLRs for BP4 (Figure 3B,C). Apart from predictions for *TERT* variants, the tools were however highly specific. The best performing tool was VEST4. All genes tested met the target PLRs for the VEST4 cut‐offs for supporting‐ and moderate‐level evidence apart from *TERT*. The lower limit of the 95% CI was also lower than 4.1 for *BRCA2* for the BP4 moderate‐level‐evidence cut‐off; however, no false positive results were seen, and further, at the cut‐off for supporting‐level evidence, the lower limit of the 95% CI was 5.43, exceeding the moderate level target PLR.

At the cut‐offs for BP4 strong level, only CADD predictions for *BRCA1*, *BRCA2* and the ClinGen groups were associated with true positive results. No false positive results were seen associated with the strong level cut‐off scores for CADD; however, poor sensitivity resulted in all 95% CIs dropping below the target of 18.7.

### 3.4. AlphaMissense Predictions

Predictive accuracy for the assessed genes was also variable. The generic cut‐off of ≥ 0.564 was associated with high specificity (> 96%) for pathogenic *BRCA1* and *BRCA2* variants whilst maintaining high sensitivity (> 88%). Predictions for benign *BRCA1* and *BRCA2* variants were also associated with high specificity (> 90%); however, sensitivity was comparatively high (> 91%). Sensitivity for benign *TP53* variants was lower (88%); however, this is compared to a median of 48% for PP3 supporting‐level predictions for the other tools assessed. The highest PLR 95% CI lower limit was associated with PP3 predictions for *BRCA1* (22.2; meeting PP3 strong). Comparatively poor sensitivity for pathogenic and benign *ATM* variants and pathogenic *TERT* variants was seen, similar to the other *in silico* tools included in this analysis (Table S10). Excluding *TERT* variants, all predictions were associated with an LR of greater than 2.1 (supporting‐level evidence) for both benign and pathogenic predictions (Table S10).

### 3.5. Optimisation of *In Silico* Cut‐Off Scores for *TERT*

In light of the suboptimal performance of gene‐agnostic *in silico* score cut‐offs for *TERT*, the ROC curves were reviewed to determine if the PLRs could be optimised. Of the tools reviewed, VEST4 was associated with the highest AUC, 0.86 (Figure 2). Sensitivity and specificity calculations were suggestive of this being predominantly due to superior specificity. Therefore, the VEST4 cut‐off scores for PP3 supporting and BP4 were revised down and up, respectively, to improve sensitivity. Revising down the VEST4 cut‐off score for PP3 supporting to 0.5 and above, the sensitivity improved to 67% and 59% for TERT_3 and TERT_1, respectively. Specificity improved to 0.9 and 1 for TERT_3 and TERT_1, respectively. The PLR 95% CI lower limit slightly improved to 2.5 and 1.4 for TERT_3 and TERT_1, respectively (Figure 3D). For BP4, a VEST4 score of equal to or below 0.16 was associated with improved sensitivity to 50% and 50%, respectively, and specificity of 90% and 97%, respectively. The PLR 95% CI lower limit was 2.2 and 2.9 for TERT_3 and TERT_1, respectively (Figure 3D). Although the gene‐agnostic predictions of pathogenicity and benignity generated by the newly released AlphaMissense [25] *in silico* tool show similarly poor PP3 sensitivity (50%) and BP4 specificity (61%) (TERT_1 dataset), a similar cut‐off adjustment also significantly improved the predictive accuracy of the tool. Applying a pathogenic prediction at a cut‐off score of greater than 0.40 and a benign prediction at a cut‐off score of less than 0.09 was associated with a PP3 sensitivity for pathogenic variants of 59% and a BP4 specificity for benign variants of 100%, respectively. PLRs also improved as shown in Figure 3D.

### 3.6. MISCAST Prediction Gene Conformance to Missense Variant–Protein Structural and Functional Impact Relationships Identified in Large Multigene Training Sets

**Figure 4:**
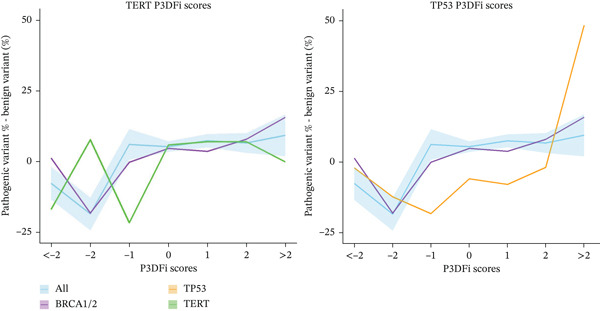
The relative enrichment of pathogenic variants at P3DF1 scores ranging from < −2 to > 2 for the genes included in this study as well as the MISCAST combined dataset of 1330 disease‐causing genes [24]. Relative enrichment of pathogenic variants is determined by the percentage of pathogenic variants minus the percentage of benign variants at each P3DFi score. Increasing P3DF1 score is associated with increasing predicted structural impact from a given missense variant. P3DFi scores of *TERT* (TERT_3) and *TP53* (TP53_2) are shown in orange and coral, respectively. Comparative P3DFi scores for the MISCAST combined dataset of 1330 disease‐causing genes are shown in blue. The standard error of the mean pathogenic variant enrichment for *ATM*, *BRCA1*, *BRCA2* and the combined MISCAST dataset of 1330 disease‐causing genes is shown by the shaded pale blue region. The mean enrichment of pathogenic variants at each P3DFi score for a combined dataset of *BRCA1* and *BRCA2* variants is shown in purple. Plots generated using gglot2, https://ggplot2.tidyverse.org.

*In silico* tools use ‘black box’ algorithms and are frequently composites of other online *in silico* tools, and the weighting to each source tool to deliver a ‘score’ for a given variant is opaque. To investigate how protein structural and functional impact predictions may be influencing *in silico* scores for the gene variants included in this study, the pathogenic 3D Feature index (P3DFi) [24] was compared with the previously examined tools. The MISCAST algorithm mines and annotates amino acid residues for structural, physiochemical and functional features to deliver a P3DFi score. Similar protein feature databases, such as UniProt [42], protein–protein interaction databases (PDBsum [64]) and protein secondary structure databases (DSSP [65]), have also been incorporated into MutPred2 [19], PolyPhen2 [66] and Mutation Assessor [67] (and therefore REVEL [18], BayesDel [20] and CADD [23]). In addition, VEST4 [21] incorporates protein feature data from these data sources via SNVBox [45]. P3DFi scores for *BRCA1*, *BRCA2* and *ATM* showed similar enrichment for pathogenic variants as the MISCAST gene variant dataset [24] (Figure 4). Lower P3DFi scores (< −1) appeared, however, to be more enriched for pathogenic *TERT* variants compared to *TP53*, *BRCA1*, *BRCA2*, *ATM* and the complete MISCAST dataset of variants from 1330 disease‐causing genes (Figure 3 and Tables S11, S12, S13, S14 and S15). Scores −1 to 2 were more enriched for benign *TP53* variants compared to the other groups, which may account for the poor sensitivity of BP4 predictions for benign and poor specificity of PP3 predictions for pathogenic *TP53* variants, respectively.

## 4. Discussion

### 4.1. Performance of *In Silico* Tools

Where possible, the ACMG/AMP guidelines [9] recommend statistical analysis of curation rules to assess if their application will result in final variant classifications with high posterior probabilities of being correct. Likely pathogenic classifications should be associated with a posterior probability of at least 0.9, and pathogenic classifications should have a posterior probability of > 0.99 [27]. Using the current approaches for combining evidence using the ACMG/AMP guidelines, a target posterior probability can then be determined for each strength level of an individual rule (supporting, moderate, strong and very strong). By sorting the scores for a combined dataset of pathogenic and benign variants from ClinVar and gnomAD, Pejaver et al. (2022) [16] were able to determine score intervals for a given *in silico* tool that would result in these predefined posterior probability targets.

At the interval cut‐offs suggested by that work, in general, the *in silico* tools assessed in this study demonstrated high specificity at the expense of sensitivity. Where tools did not meet the target PLRs, this was most commonly due to poor sensitivity. In the context of diagnostic curation, a trade‐off in favour of high specificity is appropriate as high confidence in application of PP3 or BP4 is preferable. With the exception of BP4 predictions for *TERT* and PP3 predictions for *TERT* and *TP53*, the results from this study demonstrate that the gene‐agnostic use of PP3 and BS4 at multiple strength levels is associated with acceptable specificity (at least 80% at supporting level and at least 90% at moderate level). This supports the use of this approach when there are insufficient variants with established non‐VUS classifications to perform a gene‐specific validation. These results also highlight the importance of considering specificity and sensitivity in combination with the PLR when assessing the strength of evidence a tool or ACMG/AMP rule provides. Relying on the PLR results alone may result in rejection of an *in silico* tool and score cut‐off that is highly specific if the sensitivity is low. This trade‐off is necessary due to the variability in tool performance across genes. Indeed, although this study shows that the more recently developed AlphaMissense predictions may improve sensitivity whilst preserving high specificity at least for *TP53* variants, a finding similar to other *in silico* tool evaluations for *TP53* [68], gene‐agnostic cut‐offs would likely need to be stricter if predictions are to be applied at strength levels other than supporting.

These results also show that *in silico* prediction tool performance is variable. For example, many *in silico* *TP53* BP4 and PP3 predictions were associated with comparatively poor sensitivity and poor specificity, respectively. This finding is in line with a recent performance assessment utilising ClinVar data [11]. The associated P3DFi scores are suggestive that the *TP53* protein may have increased tolerance for physicochemical changes associated with missense variants compared to other disease‐causing genes commonly included in training datasets. This is further supported by a lack of constraint for missense variants in *TP53*, even within the DNA binding domain [69, 70]. Residue location in three‐dimensional space may also be an explanation for tool performance variability. Relative solvent accessibility (RSA) predicts the location of a residue (surface or buried) within the three‐dimensional structure of a protein [46, 68]. A recent analysis of *TP53* variants annotated for RSA also demonstrated poor specificity for pathogenic buried/partially buried variants where the *ΔΔG* (structural instability) score was < 2.5 (BayesDel and AlphaMissense) [68]. When the addition of a *ΔΔG* score threshold ≥ 2.5 kcal/mol was required, the specificity of AlphaMissense scores of ≥ 0.564 for PP3 improved from 34% to 95% when assessing buried or partially buried variants. This is compared to a specificity of 73% associated with AlphaMissense predictions for *BRCA1* missense variants associated with buried/partially buried residues [46]. *In silico* predictions for *TERT* variants were the most inferior. The poor concordance seen in all *TERT* variant datasets appeared to be driven by poor sensitivity for pathogenic variants and poor specificity for benign variants. *TERT* is a highly constrained gene with less enrichment of benign variants seen in P3DFi scores of < −1. As *in silico* tools are trained using datasets of large numbers of genes, scores may be insensitive to genes encoding proteins that are highly sensitive to small physicochemical changes. Additionally, *in silico* predictions for pathogenic *TERT* variants may be less reliable when residues are surface exposed. Missense variants impacting surface‐exposed variants important for protein, RNA and DNA interaction may account for a significant subset of pathogenic *TERT* variants compared with other genes [71]. Surface‐exposed residues, however, likely account for a minority of pathogenic missense variants across the protein‐coding gene spectrum and thus may be under‐represented in training sets [72–74]. Supporting this, data available from characTERT [71] demonstrate that AlphaMissense pathogenicity predictions for exposed residues (defined as RSA > 20%) correlate poorly with CharacTERT pathogenicity predictions (sensitivity 25%) compared to predictions for buried/partially buried residues (sensitivity of 66%). Although AlphaMissense performs relatively well when assessed against MAVE data in *BRCA1*, it and BayesDel performed worse when only exposed residues are assessed [46]. Only 28% of MAVE class non‐functional variants had an AlphaMissense score of > 0.75 in this dataset. Revising cut‐off scores for the *in silico* predictor with the highest AUC (VEST4) to improve sensitivity was associated with improved PLRs for both PP3 and BP4. Similar improvements in PP3 sensitivity and BP4 specificity were also seen when AlphaMissense cut‐off scores were revised in the same direction. These results support the use of gene‐specific *in silico* tool validation where there are sufficient data to do so. Selecting different tools and cut‐offs for different genes, however, needs to be weighed up against the problems this creates for variant filtering in large gene panels and exomes prior to manual curation. Although no one tool performed the best for all genes studied in this analysis (Figure 2), a commonly used tool associated with adequate PLRs or 100% specificity following a gene‐specific validation may be appropriate for use if this simplifies bioinformatic workflows.

### 4.2. The Use of Truth Sets of Variants With Standardised Curation

*In silico* tools use a range of data associated with training sets of variants such as population variation, evolutionary conservation, physiochemical differences between reference and variant amino acids and predicted disruptions to the 3D protein structure to predict the impact of variants on protein function. The most commonly used training sets include the ClinVar, HGMD and gnomAD databases. These attribute–pathogenicity associations are therefore determined using sets of highly heterogeneous genes. These associations may not necessarily be generalisable to a given individual gene. The ClinVar and HGMD databases collate curations from submissions from individual diagnostic laboratories. The given curations are not standardised, or vetted, and minimal data are available regarding the evidence used for most individual curations, including the use of *in silico* tool evidence. Increasing efforts, by groups such as ClinGen, have been underway to standardise curations [1, 10]. Where possible, this study has utilised these guidelines to create truth sets of variants curated according to standardised approaches. This has provided the best available data to benchmark the *in silico* tools assessed in this validation study.

### 4.3. Limitations

Where possible, truth sets that contained curations with multiple applied lines of validated evidence were used in this analysis. The ClinGen, *BRCA1* and *BRCA2* curation databases provided standardised curations with detailed information on the use of evidence. *TERT* variants were also curated using a standardised approach in line with current recommendations [2, 8]. Unfortunately, at the current time, the available ClinGen curated *ATM* variants were insufficient in number for statistical analysis, and although the variants included in the LOVD3 database are collated by a single curator, complete details on the curation process are not publicly available. Where possible, truth sets were limited to variants that met a non‐VUS classification without the use of PP3 or BP4. This was possible for *TERT*, *TP53* and the *ClinGen* gene variant data. The dependency on *in silico* data for the classification of *BRCA1*, *BRCA2* and *ATM* variants could not be determined from the publicly available data. The inclusion of variants with classifications that were dependent on results from the *in silico* tools assessed in this study may have resulted in an overestimation of the accuracy of the tools for these genes. Apart from CADD [23], which was not trained on datasets of curated variants, the variants in this study may also have been included in the original training datasets for the *in silico* tools assessed. This may also have overestimated the accuracy of these *in silico* tools. Given, however, the training datasets included large numbers of different genes, it is unlikely the underlying algorithms would be strongly influenced by the individual genes assessed in this study.

## 5. Conclusions

The currently available *in silico* tools are trained on truth sets comprised of variants from multiple genes. The function of proteins such as TERT may be impacted by missense variants by mechanisms that are under‐represented in these combined datasets such as impaired/altered DNA or protein interactions. The results from this study highlight the importance of gene‐specific validation of i*in silico* tool use especially if PP3/BP4 is applied at moderate or strong levels. We have also identified that although *in silico* tool predictions are associated with high specificity, improving tool sensitivity remains an area of need.

## Author Contributions

Conceptualisation: N.N., S.L. and J.D. Data curation: N.N., A.A. and T.B. Formal analysis: N.N., A.A. and T.B. Investigation: N.N., A.A. and T.B. Methodology: N.N. and S.L. Resources: A.A. and T.B. Software: K.F. Supervision: J.D., K.F. and S.L. Validation: N.N., J.D. and T.B. Visualisation: N.N. and K.F. Writing—original draft: N.N. Writing—review and editing: N.N., T.B., K.F., S.L. and J.D. Sionne Lucas and Joanne Dickinson have contributed to the work equally and should be regarded as cofirst authors.

## Funding

This study was supported by the University of Tasmania (Annie Bishop doctoral degree scholarship), Maddie Riewoldt′s Vision, National Health and Medical Research Council (10.13039/501100000925, 2020517), Select Foundation, Arcus Foundation (10.13039/100016681), The Neil and Norma Hill Foundation and Luminesce Alliance. Open access publishing facilitated by University of Tasmania, as part of the Wiley ‐ University of Tasmania agreement via the Council of Australasian University Librarians.

## Disclosure

A preprint of this manuscript has previously been published [65].

## Ethics Statement

All genetic data analysed and reported in this study have been previously published, are discussed in articles that are in press and/or stem from publicly available databases such as ClinVar (https://www.ncbi.nlm.nih.gov/clinvar/). No institutional review board or research ethics committee approval was required.

## Conflicts of Interest

The authors declare no conflicts of interest.

## General Statement

*Web Resources.* ClinGen Evidence Repository: https://erepo.clinicalgenome.org/evrepo/; *BRCA1* and *BRCA2* mutation databases (University of Utah): https://arup.utah.edu/database/BRCA/; *ATM* gene home page; Leiden Open Variation Database (LOVD3): https://databases.lovd.nl/shared/genes/ATM; dbNSFP database: https://sites.google.com/site/jpopgen/dbNSFP; gnomAD browser: https://gnomad.broadinstitute.org/; ClinVar: https://www.ncbi.nlm.nih.gov/clinvar/; Mastermind Genomic Search Engine: https://mastermind.genomenon.com/; Variant Effect Predictor: http://grch37.ensembl.org/Homo_sapiens/Tools/VEP; MIssense variant to protein StruCture Analysis web SuiTe: https://miscast.broadinstitute.org/; ProteinPaint: https://proteinpaint.stjude.org/; Ggplot2: https://ggplot2.tidyverse.org/; https://biosig.lab.uq.edu.au/charactert.

## Supporting information


**Supporting Information** Additional supporting information can be found online in the Supporting Information section. Table S1: Variants included in Fortuno et al. (2018) (PMID 29775997), annotated with VEST4, REVEL, MutPred2, BayesDel (no AF) and CADD (phred_Hg19) scores (dbNSEP Version 4.2). Table S2: TP53 variants extracted from the ClinGen database of curated variants, annotated with VEST4, REVEL, MutPred2, BayesDel (no AF) and CADD (phred_Hg19) scores (dbNSEP Version 4.2). Table S3: BRCA1 variants extracted from the University of Utah database of curated variants, annotated with VEST4, REVEL, MutPred2, BayesDel (no AF) and CADD (phred_Hg19) scores (dbNSEP Version 4.2). Table S4: BRCA2 Variants extracted from the University of Utah database of curated variants, annotated with VEST4, REVEL, MutPred2, BayesDel (no AF) and CADD (phred_Hg19) scores (dbNSEP Version 4.2). Table S5: ATM variants extracted from the LOVD3 database of curated variants, annotated with VEST4, REVEL, MutPred2, BayesDel (no AF) and CADD (phred_Hg19) scores (dbNSEP Version 4.2). Table S6: Curated TERT variants annotated with VEST4, REVEL, MutPred2, BayesDel (no AF) and CADD (phred_Hg19) scores (dbNSEP Version 4.2). Table S7: TERT variants with published direct telomerase assay (DTA) results, annotated with VEST4, REVEL, MutPred2, BayesDel (no AF) and CADD (phred_Hg19) scores (dbNSEP Version 4.2). Table S8: Variants extracted from the ClinVar database of curated variants, annotated with VEST4, REVEL, MutPred2, BayesDel (no AF) and CADD (phred_Hg19) scores (dbNSEP Version 4.2). Table S9: Sensitivity and specificity calculations. Table S10: Sensitivity and specificity calculations for AlphaMissense. Table S11: TERT P3DFi scores extracted from MISCAST, annotated with non‐VUS curation where known. Table S12: TP53 P3DFi scores extracted from MISCAST, annotated with non‐VUS curation where known. Table S13: BRCA1 P3DFi scores extracted from MISCAST, annotated with non‐VUS curation where known. Table S14: BRCA2 P3DFi scores extracted from MISCAST, annotated with non‐VUS curation where known. Table S15: ATM P3DFi scores extracted from MISCAST, annotated with non‐VUS curation where known. Table S16: Variants included in this analysis, annotated with SpliceAI delta scores. Table S17: Variants included in this analysis with SpliceAI delta scores greater than 0.2.

## Data Availability

Additional data supporting the findings of this study are available in either the manuscript and/or the Supporting Information.

## References

[1] Luo X. , Feurstein S. , Mohan S. , Porter C. C. , Jackson S. A. , Keel S. , Chicka M. , Brown A. L. , Kesserwan C. , Agarwal A. , Luo M. , Li Z. , Ross J. E. , Baliakas P. , Pineda-Alvarez D. , DiNardo C. D. , Bertuch A. A. , Mehta N. , Vulliamy T. , Wang Y. , Nichols K. E. , Malcovati L. , Walsh M. F. , Rawlings L. H. , McWeeney S. K. , Soulier J. , Raimbault A. , Routbort M. J. , Zhang L. , Ryan G. , Speck N. A. , Plon S. E. , Wu D. , and Godley L. A. , ClinGen Myeloid Malignancy Variant Curation Expert Panel Recommendations for Germline RUNX1 Variants, Blood Advances. (2019) 3, no. 20, 2962–2979, 10.1182/bloodadvances.2019000644, 31648317. 31648317 PMC6849945

[2] Feurstein S. , Hahn C. N. , Mehta N. , and Godley L. A. , A Practical Guide to Interpreting Germline Variants That Drive Hematopoietic Malignancies, Bone Marrow Failure, and Chronic Cytopenias, Genetics in Medicine. (2022) 24, no. 4, 931–954, 10.1016/j.gim.2021.12.008, 35063349. 35063349

[3] Giacomelli A. O. , Yang X. , Lintner R. E. , McFarland J. M. , Duby M. , Kim J. , Howard T. P. , Takeda D. Y. , Ly S. H. , Kim E. , Gannon H. S. , Hurhula B. , Sharpe T. , Goodale A. , Fritchman B. , Steelman S. , Vazquez F. , Tsherniak A. , Aguirre A. J. , Doench J. G. , Piccioni F. , Roberts C. W. M. , Meyerson M. , Getz G. , Johannessen C. M. , Root D. E. , and Hahn W. C. , Mutational Processes Shape the Landscape of TP53 Mutations in Human Cancer, Nature Genetics. (2018) 50, no. 10, 1381–1387, 10.1038/s41588-018-0204-y, 30224644. 30224644 PMC6168352

[4] Kato S. , Han S. Y. , Liu W. , Otsuka K. , Shibata H. , Kanamaru R. , and Ishioka C. , Understanding the Function-Structure and Function-Mutation Relationships of p53 Tumor Suppressor Protein by High-Resolution Missense Mutation Analysis, Proceedings of the National Academy of Sciences. (2003) 100, no. 14, 8424–8429, 10.1073/pnas.1431692100, 12826609. PMC166245 12826609

[5] Kim H. K. , Lee E. J. , Lee Y. J. , Kim J. , Kim Y. , Kim K. , Lee S. W. , Chang S. , Lee Y. J. , Lee J. W. , Lee W. , Chun S. , Son B. H. , Jung K. H. , Kim Y. M. , Min W. K. , and Ahn S. H. , Impact of Proactive High-Throughput Functional Assay Data on BRCA1 Variant Interpretation in 3684 Patients With Breast or Ovarian Cancer, Journal of Human Genetics. (2020) 65, no. 3, 209–220, 10.1038/s10038-019-0713-2. 31907386

[6] Millot G. A. , Carvalho M. A. , Caputo S. M. , Vreeswijk M. P. G. , Brown M. A. , Webb M. , Rouleau E. , Neuhausen S. L. , Hansen T. O. , Galli A. , Brandão R. D. , Blok M. J. , Velkova A. , Couch F. J. , Monteiro A. N. A. , and on behalf of the ENIGMA (Evidence-based Network for the Interpretation of Germline Mutant Alleles) Consortium Functional Assay Working Group , A Guide for Functional Analysis of BRCA1 Variants of Uncertain Significance, Human Mutation. (2012) 33, no. 11, 1526–1537, 10.1002/humu.22150, 22753008. 22753008 PMC3470782

[7] Ikegami M. , Kohsaka S. , Ueno T. , Momozawa Y. , Inoue S. , Tamura K. , Shimomura A. , Hosoya N. , Kobayashi H. , Tanaka S. , and Mano H. , High-Throughput Functional Evaluation of BRCA2 Variants of Unknown Significance, Nature Communications. (2020) 11, no. 1, 10.1038/s41467-020-16141-8, 32444794. PMC7244490 32444794

[8] Nelson N. , Feurstein S. , Niaz A. , Truong J. , Holien J. K. , Lucas S. , Fairfax K. , Dickinson J. , and Bryan T. M. , Functional Genomics for Curation of Variants in Telomere Biology Disorder Associated Genes: A Systematic Review, Genetics in Medicine. (2023) 25, no. 3, 100354, 10.1016/j.gim.2022.11.021. 36496180

[9] Richards S. , Aziz N. , Bale S. , Bick D. , das S. , Gastier-Foster J. , Grody W. W. , Hegde M. , Lyon E. , Spector E. , Voelkerding K. , Rehm H. L. , and ACMG Laboratory Quality Assurance Committee , Standards and Guidelines for the Interpretation of Sequence Variants: A Joint Consensus Recommendation of the American College of Medical Genetics and Genomics and the Association for Molecular Pathology, Genetics in medicine. (2015) 17, no. 5, 405–424, 10.1038/gim.2015.30, 25741868. 25741868 PMC4544753

[10] Fortuno C. , Lee K. , Olivier M. , Pesaran T. , Mai P. L. , Andrade K. C. , Attardi L. D. , Crowley S. , Evans D. G. , Feng B. J. , Foreman A. K. M. , Frone M. N. , Huether R. , James P. A. , McGoldrick K. , Mester J. , Seifert B. A. , Slavin T. P. , Witkowski L. , Zhang L. , Plon S. E. , Spurdle A. B. , Savage S. A. , and the ClinGen TP53 Variant Curation Expert Panel , Specifications of the ACMG/AMP Variant Interpretation Guidelines for Germline TP53 Variants, Human Mutation. (2021) 42, no. 3, 223–236, 10.1002/humu.24152, 33300245. 33300245 PMC8374922

[11] Tejura M. , Fayer S. , McEwen A. E. , Flynn J. , Starita L. M. , and Fowler D. M. , Calibration of Variant Effect Predictors on Genome-Wide Data Masks Heterogeneous Performance Across Genes, American Journal of Human Genetics. (2024) 111, no. 9, 2031–2043, 10.1016/j.ajhg.2024.07.018, 39173626. 39173626 PMC11393694

[12] Richardson M. E. , Holdren M. , Brannan T. , de la Hoya M. , Spurdle A. B. , Tavtigian S. V. , Young C. C. , Zec L. , Hiraki S. , Anderson M. J. , Walker L. C. , McNulty S. , Turnbull C. , Tischkowitz M. , Schon K. , Slavin T. , Foulkes W. D. , Cline M. , Monteiro A. N. , Pesaran T. , and Couch F. J. , Specifications of the ACMG/AMP Variant Curation Guidelines for the Analysis of Germline ATM Sequence Variants, American Journal of Human Genetics. (2024) 111, no. 11, 2411–2426, 10.1016/j.ajhg.2024.08.022, 39317201. 39317201 PMC11568761

[13] Richardson M. E. , Bishop M. F. H. , Holdren M. A. , de la Hoya M. , Spurdle A. B. , Tavtigian S. V. , Brannan T. , Young C. C. , Zec L. , Hiraki S. , Turnbull C. , Tischkowitz M. , Bernstein K. A. , Masson J. Y. , McNulty S. M. , Pesaran T. , Monteiro A. N. , Walker L. C. , Foulkes W. D. , and Couch F. J. , Specifications of the ACMG/AMP Variant Curation Guidelines for the Analysis of Germline PALB2 Sequence Variants, American Journal of Human Genetics. (2025) 112, no. 10, 2266–2280, 10.1016/j.ajhg.2025.08.020, 40967221. 40967221 PMC12520758

[14] Lee K. , Krempely K. , Roberts M. E. , Anderson M. J. , Carneiro F. , Chao E. , Dixon K. , Figueiredo J. , Ghosh R. , Huntsman D. , Kaurah P. , Kesserwan C. , Landrith T. , Li S. , Mensenkamp A. R. , Oliveira C. , Pardo C. , Pesaran T. , Richardson M. , Slavin T. P. , Spurdle A. B. , Trapp M. , Witkowski L. , Yi C. S. , Zhang L. , Plon S. E. , Schrader K. A. , and Karam R. , Specifications of the ACMG/AMP Variant Curation Guidelines for the Analysis of germlineCDH1sequence Variants, Human Mutation. (2018) 39, no. 11, 1553–1568, 10.1002/humu.23650. 30311375 PMC6188664

[15] Spier I. , Yin X. , Richardson M. , Pineda M. , Laner A. , Ritter D. , Boyle J. , Mur P. , Hansen T. V. O. , Shi X. , Mahmood K. , Plazzer J. P. , Ognedal E. , Nordling M. , Farrington S. M. , Yamamoto G. , Baert-Desurmont S. , Martins A. , Borras E. , Tops C. , Webb E. , Beshay V. , Genuardi M. , Pesaran T. , Capellá G. , Tavtigian S. V. , Latchford A. , Frayling I. M. , Plon S. E. , Greenblatt M. , Macrae F. A. , Aretz S. , and InSiGHT-ClinGen Hereditary Colon Cancer/Polyposis Variant Curation Expert Panel , Gene-Specific ACMG/AMP Classification Criteria for Germline APC Variants: Recommendations From the ClinGen InSiGHT Hereditary Colorectal Cancer/Polyposis Variant Curation Expert Panel, Genetics in Medicine. (2024) 26, no. 2, 100992, 10.1016/j.gim.2023.100992, 37800450. 37800450 PMC10922469

[16] Pejaver V. , Byrne A. B. , Feng B. J. , Pagel K. A. , Mooney S. D. , Karchin R. , O’Donnell-Luria A. , Harrison S. M. , Tavtigian S. V. , Greenblatt M. S. , Biesecker L. G. , Radivojac P. , Brenner S. E. , Biesecker L. G. , Harrison S. M. , Tayoun A. A. , Berg J. S. , Brenner S. E. , Cutting G. R. , Ellard S. , Greenblatt M. S. , Kang P. , Karbassi I. , Karchin R. , Mester J. , O’Donnell-Luria A. , Pesaran T. , Plon S. E. , Rehm H. L. , Strande N. T. , Tavtigian S. V. , and Topper S. , Calibration of Computational Tools for Missense Variant Pathogenicity Classification and ClinGen Recommendations for PP3/BP4 Criteria, American Journal of Human Genetics. (2022) 109, no. 12, 2163–2177, 10.1016/j.ajhg.2022.10.013, 36413997. 36413997 PMC9748256

[17] Wilcox E. H. , Sarmady M. , Wulf B. , Wright M. W. , Rehm H. L. , Biesecker L. G. , and Abou Tayoun A. N. , Evaluating the Impact of In Silico Predictors on Clinical Variant Classification, Genetics in Medicine. (2022) 24, no. 4, 924–930, 10.1016/j.gim.2021.11.018, 34955381. 34955381 PMC9164215

[18] Ioannidis N. M. , Rothstein J. H. , Pejaver V. , Middha S. , McDonnell S. K. , Baheti S. , Musolf A. , Li Q. , Holzinger E. , Karyadi D. , Cannon-Albright L. A. , Teerlink C. C. , Stanford J. L. , Isaacs W. B. , Xu J. , Cooney K. A. , Lange E. M. , Schleutker J. , Carpten J. D. , Powell I. J. , Cussenot O. , Cancel-Tassin G. , Giles G. G. , MacInnis R. J. , Maier C. , Hsieh C. L. , Wiklund F. , Catalona W. J. , Foulkes W. D. , Mandal D. , Eeles R. A. , Kote-Jarai Z. , Bustamante C. D. , Schaid D. J. , Hastie T. , Ostrander E. A. , Bailey-Wilson J. E. , Radivojac P. , Thibodeau S. N. , Whittemore A. S. , and Sieh W. , REVEL: An Ensemble Method for Predicting the Pathogenicity of Rare Missense Variants, American Journal of Human Genetics. (2016) 99, no. 4, 877–885, 10.1016/j.ajhg.2016.08.016, 27666373. 27666373 PMC5065685

[19] Pejaver V. , Urresti J. , Lugo-Martinez J. , Pagel K. A. , Lin G. N. , Nam H. J. , Mort M. , Cooper D. N. , Sebat J. , Iakoucheva L. M. , Mooney S. D. , and Radivojac P. , Inferring the Molecular and Phenotypic Impact of Amino Acid Variants With MutPred2, Nature Communications. (2020) 11, no. 1, 10.1038/s41467-020-19669-x, 33219223. PMC7680112 33219223

[20] Feng B. J. , PERCH: A Unified Framework for Disease Gene Prioritization, Human Mutation. (2017) 38, no. 3, 243–251, 10.1002/humu.23158, 27995669. 27995669 PMC5299048

[21] Douville C. , Masica D. L. , Stenson P. D. , Cooper D. N. , Gygax D. M. , Kim R. , Ryan M. , and Karchin R. , Assessing the Pathogenicity of Insertion and Deletion Variants With the Variant Effect Scoring Tool (VEST-Indel), Human Mutation. (2016) 37, no. 1, 28–35, 10.1002/humu.22911, 26442818. 26442818 PMC5057310

[22] Rentzsch P. , Schubach M. , Shendure J. , and Kircher M. , CADD-Splice-Improving Genome-Wide Variant Effect Prediction Using Deep Learning-Derived Splice Scores, Genome Medicine. (2021) 13, no. 1, 10.1186/s13073-021-00835-9, 33618777. PMC7901104 33618777

[23] Rentzsch P. , Witten D. , Cooper G. M. , Shendure J. , and Kircher M. , CADD: Predicting the Deleteriousness of Variants Throughout the Human Genome, Nucleic Acids Res. (2019) 47, no. D1, D886–D894, 10.1093/nar/gky1016, 30371827. 30371827 PMC6323892

[24] Iqbal S. , Perez-Palma E. , Jespersen J. B. , May P. , Hoksza D. , Heyne H. O. , Ahmed S. S. , Rifat Z. T. , Rahman M. S. , Lage K. , and Palotie A. , Comprehensive Characterization of Amino Acid Positions in Protein Structures Reveals Molecular Effect of Missense Variants, Proceedings of the National Academy of Sciences. (2020) 117, no. 45, 28201–28211, 10.1073/pnas.2002660117, 33106425. PMC7668189 33106425

[25] Cheng J. , Novati G. , Pan J. , Bycroft C. , Žemgulytė A. , Applebaum T. , Pritzel A. , Wong L. H. , Zielinski M. , Sargeant T. , Schneider R. G. , Senior A. W. , Jumper J. , Hassabis D. , Kohli P. , and Avsec Ž. , Accurate Proteome-Wide Missense Variant Effect Prediction With AlphaMissense, Science. (2023) 381, no. 6664, eadg7492, 10.1126/science.adg7492, 37733863. 37733863

[26] Brnich S. E. , Abou Tayoun A. N. , Couch F. J. , Cutting G. R. , Greenblatt M. S. , Heinen C. D. , Kanavy D. M. , Luo X. , McNulty S. , Starita L. M. , Tavtigian S. V. , Wright M. W. , Harrison S. M. , Biesecker L. G. , Berg J. S. , and Clinical Genome Resource Sequence Variant Interpretation Working Group , Recommendations for Application of the Functional Evidence PS3/BS3 Criterion Using the ACMG/AMP Sequence Variant Interpretation Framework, Genome Medicine. (2019) 12, no. 1, 10.1186/s13073-019-0690-2, 31892348. PMC6938631 31892348

[27] Tavtigian S. V. , Greenblatt M. S. , Harrison S. M. , Nussbaum R. L. , Prabhu S. A. , Boucher K. M. , Biesecker L. G. , and ClinGen Sequence Variant Interpretation Working Group (ClinGen SVI) , Modeling the ACMG/AMP Variant Classification Guidelines as a Bayesian Classification Framework, Genome medicine. (2018) 20, no. 9, 1054–1060, 10.1038/gim.2017.210, 29300386. PMC6336098 29300386

[28] Reva B. , Antipin Y. , and Sander C. , Predicting the Functional Impact of Protein Mutations: Application to Cancer Genomics, Nucleic Acids Research. (2011) 39, no. 17, e118, 10.1093/nar/gkr407, 21727090. 21727090 PMC3177186

[29] Schwarz J. M. , Rodelsperger C. , Schuelke M. , and Seelow D. , MutationTaster Evaluates Disease-Causing Potential of Sequence Alterations, Nature Methods. (2010) 7, no. 8, 575–576, 10.1038/nmeth0810-575, 20676075. 20676075

[30] Adzhubei I. A. , Schmidt S. , Peshkin L. , Ramensky V. E. , Gerasimova A. , Bork P. , Kondrashov A. S. , and Sunyaev S. R. , A Method and Server for Predicting Damaging Missense Mutations, Nature Methods. (2010) 7, no. 4, 248–249, 10.1038/nmeth0410-248, 20354512. 20354512 PMC2855889

[31] Chun S. and Fay J. C. , Identification of Deleterious Mutations Within Three Human Genomes, Genome Research. (2009) 19, no. 9, 1553–1561, 10.1101/gr.092619.109, 19602639. 19602639 PMC2752137

[32] Kumar P. , Henikoff S. , and Ng P. C. , Predicting the Effects of Coding Non-Synonymous Variants on Protein Function Using the SIFT Algorithm, Nature Protocols. (2009) 4, no. 7, 1073–1081, 10.1038/nprot.2009.86, 19561590. 19561590

[33] Davydov E. V. , Goode D. L. , Sirota M. , Cooper G. M. , Sidow A. , and Batzoglou S. , Identifying a High Fraction of the Human Genome to Be Under Selective Constraint Using GERP++, PLoS Computational Biology. (2010) 6, no. 12, e1001025, 10.1371/journal.pcbi.1001025, 21152010. 21152010 PMC2996323

[34] Pollard K. S. , Hubisz M. J. , Rosenbloom K. R. , and Siepel A. , Detection of Nonneutral Substitution Rates on Mammalian Phylogenies, Genome Research. (2010) 20, no. 1, 110–121, 10.1101/gr.097857.109, 19858363. 19858363 PMC2798823

[35] Garber M. , Guttman M. , Clamp M. , Zody M. C. , Friedman N. , and Xie X. , Identifying Novel Constrained Elements by Exploiting Biased Substitution Patterns, Bioinformatics. (2009) 25, no. 12, i54–i62, 10.1093/bioinformatics/btp190, 19478016. 19478016 PMC2687944

[36] Shihab H. A. , Gough J. , Cooper D. N. , Stenson P. D. , Barker G. L. A. , Edwards K. J. , Day I. N. M. , and Gaunt T. R. , Predicting the Functional, Molecular, and Phenotypic Consequences of Amino Acid Substitutions Using Hidden Markov Models, Human Mutation. (2013) 34, no. 1, 57–65, 10.1002/humu.22225, 23033316. 23033316 PMC3558800

[37] Choi Y. , Sims G. E. , Murphy S. , Miller J. R. , and Chan A. P. , Predicting the Functional Effect of Amino Acid Substitutions and Indels, PLoS One. (2012) 7, no. 10, e46688, 10.1371/journal.pone.0046688, 23056405. 23056405 PMC3466303

[38] Carter H. , Douville C. , Stenson P. D. , Cooper D. N. , and Karchin R. , Identifying Mendelian Disease Genes With the Variant Effect Scoring Tool, BMC Genomics. (2013) 14, no. S3, 10.1186/1471-2164-14-S3-S3. PMC3665549 23819870

[39] Landrum M. J. , Lee J. M. , Benson M. , Brown G. R. , Chao C. , Chitipiralla S. , Gu B. , Hart J. , Hoffman D. , Jang W. , Karapetyan K. , Katz K. , Liu C. , Maddipatla Z. , Malheiro A. , McDaniel K. , Ovetsky M. , Riley G. , Zhou G. , Holmes J. B. , Kattman B. L. , and Maglott D. R. , ClinVar: Improving Access to Variant Interpretations and Supporting Evidence, Nucleic Acids Research. (2018) 46, no. D1, D1062–D1067, 10.1093/nar/gkx1153, 29165669. 29165669 PMC5753237

[40] Stenson P. D. , Ball E. V. , Mort M. , Phillips A. D. , Shiel J. A. , Thomas N. S. T. , Abeysinghe S. , Krawczak M. , and Cooper D. N. , Human Gene Mutation Database (HGMD): 2003 Update, Human Mutation. (2003) 21, no. 6, 577–581, 10.1002/humu.10212, 12754702. 12754702

[41] Lek M. , Karczewski K. J. , Minikel E. V. , Samocha K. E. , Banks E. , Fennell T. , O’Donnell-Luria A. H. , Ware J. S. , and Hill A. J. , Analysis of Protein-Coding Genetic Variation in 60,706 Humans, Nature. (2016) 536, no. 7616, 285–291, 10.1038/nature19057, 27535533. 27535533 PMC5018207

[42] UniProt C. , UniProt: the Universal Protein Knowledgebase in 2023, Nucleic Acids Research. (2023) 51, no. D1, D523–D531. 36408920 10.1093/nar/gkac1052 PMC9825514

[43] Laskowski R. A. , Jablonska J. , Pravda L. , Varekova R. S. , and Thornton J. M. , PDBsum: Structural Summaries of PDB Entries, Protein Science. (2018) 27, no. 1, 129–134, 10.1002/pro.3289, 28875543. 28875543 PMC5734310

[44] Fu W. , O’Connor T. D. , Jun G. , Kang H. M. , Abecasis G. , Leal S. M. , Gabriel S. , Rieder M. J. , Altshuler D. , Shendure J. , Nickerson D. A. , Bamshad M. J. , NHLBI Exome Sequencing Project , and Akey J. M. , Analysis of 6,515 Exomes Reveals the Recent Origin of Most Human Protein-Coding Variants, Nature. (2013) 493, no. 7431, 216–220, 10.1038/nature11690, 23201682. 23201682 PMC3676746

[45] Wong W. C. , Kim D. , Carter H. , Diekhans M. , Ryan M. C. , and Karchin R. , CHASM and SNVBox: Toolkit for Detecting Biologically Important Single Nucleotide Mutations in Cancer, Bioinformatics. (2011) 27, no. 15, 2147–2148, 10.1093/bioinformatics/btr357, 21685053. 21685053 PMC3137226

[46] Ramadane-Morchadi L. , Rotenberg N. , Esteban-Sanchez A. , Fortuno C. , Gómez-Sanz A. , Varga M. J. , Chamberlin A. , Richardson M. E. , Michailidou K. , Pérez-Segura P. , and Spurdle A. B. , ACMG/AMP Interpretation of BRCA1 Missense Variants: Structure-Informed Scores Add Evidence Strength Granularity to the PP3/BP4 Computational Evidence, American Journal of Human Genetics. (2025) 112, no. 5, 993–1002, 10.1016/j.ajhg.2024.12.011, 40233743. 40233743 PMC12120176

[47] Jaganathan K. , Kyriazopoulou Panagiotopoulou S. , McRae J. F. , Darbandi S. F. , Knowles D. , Li Y. I. , Kosmicki J. A. , Arbelaez J. , Cui W. , Schwartz G. B. , Chow E. D. , Kanterakis E. , Gao H. , Kia A. , Batzoglou S. , Sanders S. J. , and Farh K. K. H. , Predicting Splicing From Primary Sequence With Deep Learning, Cell. (2019) 176, no. 3, 535–548.e24, 10.1016/j.cell.2018.12.015, 30661751. 30661751

[48] Bejar R. , Stevenson K. , Abdel-Wahab O. , Galili N. , Nilsson B. , Garcia-Manero G. , Kantarjian H. , Raza A. , Levine R. L. , Neuberg D. , and Ebert B. L. , Clinical Effect of Point Mutations in Myelodysplastic Syndromes, New England Journal of Medicine. (2011) 364, no. 26, 2496–2506, 10.1056/NEJMoa1013343, 21714648. 21714648 PMC3159042

[49] Feurstein S. , Adegunsoye A. , Mojsilovic D. , Vij R. , West DePersia A. H. , Rajagopal P. S. , Osman A. , Collins R. H. , Kim R. H. , Gore S. D. , Greenberg P. , Godley L. A. , Li Z. , del Gaudio D. , Subramanian H. P. , das S. , Walsh T. , Gulsuner S. , Segal J. P. , Husain A. N. , Gurbuxani S. , King M. C. , Strek M. E. , and Churpek J. E. , Telomere Biology Disorder Prevalence and Phenotypes in Adults With Familial Hematologic and/or Pulmonary Presentations, Blood Advances. (2020) 4, no. 19, 4873–4886, 10.1182/bloodadvances.2020001721, 33035329. 33035329 PMC7556157

[50] Walker L. C. , Hoya M. , Wiggins G. A. R. , Lindy A. , Vincent L. M. , Parsons M. T. , Canson D. M. , Bis-Brewer D. , Cass A. , Tchourbanov A. , Zimmermann H. , Byrne A. B. , Pesaran T. , Karam R. , Harrison S. M. , Spurdle A. B. , Biesecker L. G. , Harrison S. M. , Tayoun A. A. , Berg J. S. , Brenner S. E. , Cutting G. R. , Ellard S. , Greenblatt M. S. , Kang P. , Karbassi I. , Karchin R. , Mester J. , O’Donnell-Luria A. , Pesaran T. , Plon S. E. , Rehm H. L. , Strande N. T. , Tavtigian S. V. , and Topper S. , Using the ACMG/AMP Framework to Capture Evidence Related to Predicted and Observed Impact on Splicing: Recommendations From the ClinGen SVI Splicing Subgroup, American Journal of Human Genetics. (2023) 110, no. 7, 1046–1067, 10.1016/j.ajhg.2023.06.002, 37352859. 37352859 PMC10357475

[51] DiStefano M. T. , Goehringer S. , Babb L. , Alkuraya F. S. , Amberger J. , Amin M. , Austin-Tse C. , Balzotti M. , Berg J. S. , Birney E. , Bocchini C. , Bruford E. A. , Coffey A. J. , Collins H. , Cunningham F. , Daugherty L. C. , Einhorn Y. , Firth H. V. , Fitzpatrick D. R. , Foulger R. E. , Goldstein J. , Hamosh A. , Hurles M. R. , Leigh S. E. , Leong I. U. S. , Maddirevula S. , Martin C. L. , McDonagh E. M. , Olry A. , Puzriakova A. , Radtke K. , Ramos E. M. , Rath A. , Riggs E. R. , Roberts A. M. , Rodwell C. , Snow C. , Stark Z. , Tahiliani J. , Tweedie S. , Ware J. S. , Weller P. , Williams E. , Wright C. F. , Yates T. M. , and Rehm H. L. , The Gene Curation Coalition: A Global Effort to Harmonize Gene-Disease Evidence Resources, Genetics in Medicine. (2022) 24, no. 8, 1732–1742, 10.1016/j.gim.2022.04.017, 35507016. 35507016 PMC7613247

[52] Marill K. A. , Chang Y. , Wong K. F. , and Friedman A. B. , Estimating Negative Likelihood Ratio Confidence When Test Sensitivity Is 100%: A Bootstrapping Approach, Statistical Methods in Medical Research. (2017) 26, no. 4, 1936–1948. 26152746 10.1177/0962280215592907

[53] Preston C. G. , Wright M. W. , Madhavrao R. , Harrison S. M. , Goldstein J. L. , Luo X. , Wand H. , Wulf B. , Cheung G. , Mandell M. E. , Tong H. , Cheng S. , Iacocca M. A. , Pineda A. L. , Popejoy A. B. , Dalton K. , Zhen J. , Dwight S. S. , Babb L. , DiStefano M. , O’Daniel J. M. , Lee K. , Riggs E. R. , Zastrow D. B. , Mester J. L. , Ritter D. I. , Patel R. Y. , Subramanian S. L. , Milosavljevic A. , Berg J. S. , Rehm H. L. , Plon S. E. , Cherry J. M. , Bustamante C. D. , Costa H. A. , and on behalf of the Clinical Genome Resource (ClinGen) , ClinGen Variant Curation Interface: A Variant Classification Platform for the Application of Evidence Criteria From ACMG/AMP Guidelines, Genome Medicine. (2022) 14, no. 1, 10.1186/s13073-021-01004-8, 35039090. PMC8764818 35039090

[54] Rehm H. L. , Berg J. S. , Brooks L. D. , Bustamante C. D. , Evans J. P. , Landrum M. J. , Ledbetter D. H. , Maglott D. R. , Martin C. L. , Nussbaum R. L. , Plon S. E. , Ramos E. M. , Sherry S. T. , Watson M. S. , and ClinGen , ClinGen—The Clinical Genome Resource, New England Journal of Medicine. (2015) 372, no. 23, 2235–2242, 10.1056/NEJMsr1406261, 26014595. 26014595 PMC4474187

[55] Fortuno C. , James P. A. , Young E. L. , Feng B. , Olivier M. , Pesaran T. , Tavtigian S. V. , and Spurdle A. B. , Improved, ACMG-Compliant, In Silico Prediction of Pathogenicity for Missense Substitutions Encoded by TP53 Variants, Human mutation. (2018) 39, no. 8, 1061–1069, 10.1002/humu.23553, 29775997. 29775997 PMC6043381

[56] Liu X. , Li C. , Mou C. , Dong Y. , and Tu Y. , dbNSFP v4: A Comprehensive Database of Transcript-Specific Functional Predictions and Annotations for Human Nonsynonymous and Splice-Site SNVs, Genome Medical. (2020) 12, no. 1, 10.1186/s13073-020-00803-9, 33261662. PMC7709417 33261662

[57] Easton D. F. , Deffenbaugh A. M. , Pruss D. , Frye C. , Wenstrup R. J. , Allen-Brady K. , Tavtigian S. V. , Monteiro A. N. A. , Iversen E. S. , Couch F. J. , and Goldgar D. E. , A Systematic Genetic Assessment of 1,433 Sequence Variants of Unknown Clinical Significance in the BRCA1 and BRCA2 Breast Cancer-Predisposition Genes, American Journal of Human Genetics. (2007) 81, no. 5, 873–883, 10.1086/521032, 17924331. 17924331 PMC2265654

[58] Sweet K. , Senter L. , Pilarski R. , Wei L. , and Toland A. E. , Characterization of BRCA1 Ring Finger Variants of Uncertain Significance, Breast Cancer Research and Treatment. (2010) 119, no. 3, 737–743, 10.1007/s10549-009-0438-6, 19543972. 19543972 PMC4283813

[59] Fokkema I. , Kroon M. , Lopez Hernandez J. A. , Asscheman D. , Lugtenburg I. , Hoogenboom J. , and den Dunnen J. T. , The LOVD3 Platform: Efficient Genome-Wide Sharing of Genetic Variants, European Journal of Human Genetics. (2021) 29, no. 12, 1796–1803, 10.1038/s41431-021-00959-x, 34521998. 34521998 PMC8632977

[60] Lefter M. , Vis J. K. , Vermaat M. , den Dunnen J. T. , Taschner P. E. M. , and Laros J. F. J. , Mutalyzer 2: Next Generation HGVS Nomenclature Checker, Bioinformatics. (2021) 37, no. 18, 2811–2817, 10.1093/bioinformatics/btab051, 33538839. 33538839 PMC8479679

[61] Sachs M. C. , plotROC: A Tool for Plotting ROC Curves, Journal of Statistical Software. (2017) 79, 10.18637/jss.v079.c02, 30686944. PMC6347406 30686944

[62] Zhou X. , Edmonson M. N. , Wilkinson M. R. , Patel A. , Wu G. , Liu Y. , Li Y. , Zhang Z. , Rusch M. C. , Parker M. , Becksfort J. , Downing J. R. , and Zhang J. , Exploring Genomic Alteration in Pediatric Cancer Using ProteinPaint, Nature Genetics. (2016) 48, no. 1, 4–6, 10.1038/ng.3466, 26711108. 26711108 PMC4892362

[63] Robin X. , Turck N. , Hainard A. , Tiberti N. , Lisacek F. , Sanchez J. C. , and Müller M. , pROC: An Open-Source Package for R and S+ to Analyze and Compare ROC Curves, BMC Bioinformatics. (2011) 12, no. 1, 10.1186/1471-2105-12-77, 21414208. PMC3068975 21414208

[64] Laskowski R. A. , Hutchinson E. G. , Michie A. D. , Wallace A. C. , Jones M. L. , and Thornton J. M. , PDBsum: A Web-Based Database of Summaries and Analyses of All PDB Structures, Trends in Biochemical Sciences. (1997) 22, no. 12, 488–490, 10.1016/S0968-0004(97)01140-7, 9433130. 9433130

[65] Touw W. G. , Baakman C. , Black J. , te Beek T. A. H. , Krieger E. , Joosten R. P. , and Vriend G. , A Series of PDB-Related Databanks for Everyday Needs, Nucleic Acids Research. (2015) 43, no. D1, D364–D368, 10.1093/nar/gku1028, 25352545. 25352545 PMC4383885

[66] Adzhubei I. , Jordan D. M. , and Sunyaev S. R. , Predicting Functional Effect of Human Missense Mutations Using PolyPhen-2, Current Protocols in Human Genetics. (2013) 76, no. 1, 7–20, 10.1002/0471142905.hg0720s76. PMC4480630 23315928

[67] Reva B. , Antipin Y. , and Sander C. , Determinants of Protein Function Revealed by Combinatorial Entropy Optimization, Genome Biology. (2007) 8, no. 11, 10.1186/gb-2007-8-11-r232, 17976239. PMC2258190 17976239

[68] Rotenberg N. , Fortuno C. , Varga M. J. , Chamberlin A. C. , Ramadane-Morchadi L. , Feng B. J. , de la Hoya M. , Richardson M. E. , and Spurdle A. B. , Integration of Protein Stability and AlphaMissense Scores Improves Bioinformatic Impact Prediction for p53 Missense and In-Frame Amino Acid Deletion Variants, American Journal of Human Genetics. (2025) 112, no. 5, 1003–1014, 10.1016/j.ajhg.2025.01.012, 40233742. 40233742 PMC12120181

[69] Karczewski K. J. , Francioli L. C. , Tiao G. , Cummings B. B. , Alföldi J. , Wang Q. , Collins R. L. , Laricchia K. M. , Ganna A. , Birnbaum D. P. , Gauthier L. D. , Brand H. , Solomonson M. , Watts N. A. , Rhodes D. , Singer-Berk M. , England E. M. , Seaby E. G. , Kosmicki J. A. , Walters R. K. , Tashman K. , Farjoun Y. , Banks E. , Poterba T. , Wang A. , Seed C. , Whiffin N. , Chong J. X. , Samocha K. E. , Pierce-Hoffman E. , Zappala Z. , O’Donnell-Luria A. H. , Minikel E. V. , Weisburd B. , Lek M. , Ware J. S. , Vittal C. , Armean I. M. , Bergelson L. , Cibulskis K. , Connolly K. M. , Covarrubias M. , Donnelly S. , Ferriera S. , Gabriel S. , Gentry J. , Gupta N. , Jeandet T. , Kaplan D. , Llanwarne C. , Munshi R. , Novod S. , Petrillo N. , Roazen D. , Ruano-Rubio V. , Saltzman A. , Schleicher M. , Soto J. , Tibbetts K. , Tolonen C. , Wade G. , Talkowski M. E. , Genome Aggregation Database Consortium , Aguilar Salinas C. A. , Ahmad T. , Albert C. M. , Ardissino D. , Atzmon G. , Barnard J. , Beaugerie L. , Benjamin E. J. , Boehnke M. , Bonnycastle L. L. , Bottinger E. P. , Bowden D. W. , Bown M. J. , Chambers J. C. , Chan J. C. , Chasman D. , Cho J. , Chung M. K. , Cohen B. , Correa A. , Dabelea D. , Daly M. J. , Darbar D. , Duggirala R. , Dupuis J. , Ellinor P. T. , Elosua R. , Erdmann J. , Esko T. , Färkkilä M. , Florez J. , Franke A. , Getz G. , Glaser B. , Glatt S. J. , Goldstein D. , Gonzalez C. , Groop L. , Haiman C. , Hanis C. , Harms M. , Hiltunen M. , Holi M. M. , Hultman C. M. , Kallela M. , Kaprio J. , Kathiresan S. , Kim B. J. , Kim Y. J. , Kirov G. , Kooner J. , Koskinen S. , Krumholz H. M. , Kugathasan S. , Kwak S. H. , Laakso M. , Lehtimäki T. , Loos R. J. F. , Lubitz S. A. , Ma R. C. W. , MacArthur D. G. , Marrugat J. , Mattila K. M. , McCarroll S. , McCarthy M. I. , McGovern D. , McPherson R. , Meigs J. B. , Melander O. , Metspalu A. , Neale B. M. , Nilsson P. M. , O’Donovan M. C. , Ongur D. , Orozco L. , Owen M. J. , Palmer C. N. A. , Palotie A. , Park K. S. , Pato C. , Pulver A. E. , Rahman N. , Remes A. M. , Rioux J. D. , Ripatti S. , Roden D. M. , Saleheen D. , Salomaa V. , Samani N. J. , Scharf J. , Schunkert H. , Shoemaker M. B. , Sklar P. , Soininen H. , Sokol H. , Spector T. , Sullivan P. F. , Suvisaari J. , Tai E. S. , Teo Y. Y. , Tiinamaija T. , Tsuang M. , Turner D. , Tusie-Luna T. , Vartiainen E. , Vawter M. P. , Ware J. S. , Watkins H. , Weersma R. K. , Wessman M. , Wilson J. G. , Xavier R. J. , Neale B. M. , Daly M. J. , and MacArthur D. G. , The Mutational Constraint Spectrum Quantified From Variation in 141,456 Humans, Nature. (2020) 581, no. 7809, 434–443, 10.1038/s41586-020-2308-7, 32461654. 32461654 PMC7334197

[70] Hayeck T. J. , Stong N. , Wolock C. J. , Copeland B. , Kamalakaran S. , Goldstein D. B. , and Allen A. S. , Improved Pathogenic Variant Localization via a Hierarchical Model of Sub-Regional Intolerance, American Journal of Human Genetics. (2019) 104, no. 2, 299–309, 10.1016/j.ajhg.2018.12.020, 30686509. 30686509 PMC6369453

[71] Georgina Becerra Parra Q. P. , Myung Y. , Portelli S. , Nelson N. E. , Dickinson J. L. , Lucas S. E. M. , Holien J. K. , Bryan T. M. , and Ascher D. B. , A Machine Learning Tool for Classifying hTERT Missense Variants, 2026, bioRxiv 10.64898/2026.05.18.725793.

[72] Varga M. J. , Richardson M. E. , and Chamberlin A. , Structural Biology in Variant Interpretation: Perspectives and Practices From Two Studies, American Journal of Human Genetics. (2025) 112, no. 5, 984–992, 10.1016/j.ajhg.2025.03.010, 40233741. 40233741 PMC12120175

[73] Livesey B. J. and Marsh J. A. , The Properties of Human Disease Mutations at Protein Interfaces, PLOS Computational Biology. (2022) 18, no. 2, e1009858, 10.1371/journal.pcbi.1009858, 35120134. 35120134 PMC8849535

[74] Vacic V. , Markwick P. R. , Oldfield C. J. , Zhao X. , Haynes C. , Uversky V. N. , and Iakoucheva L. M. , Disease-Associated Mutations Disrupt Functionally Important Regions of Intrinsic Protein Disorder, PLOS Computational Biology. (2012) 8, no. 10, e1002709, 10.1371/journal.pcbi.1002709, 23055912. 23055912 PMC3464192

